# A PC-based titrator for flow gradient titrations

**DOI:** 10.1155/S1463924693000276

**Published:** 1993

**Authors:** B. Fuhrmann, U. Spohn

**Affiliations:** Martin-Luther-Universität Halle-Wittenberg Institut für Biotechnologie Weinbergweg 16a Halle/Saale 06120 Germany

## Abstract

This paper describes a PC (personal computer) based titrator which was developed for gradient flow titrations. Concentration gradients were generated electrolytically or volumetrically in small tubes. Complete titration curves can be recorded on-line and evaluated automatically. The titrator can be used with all liquid flow detectors with low axial dispersion. The titrator was evaluated for the titration of thiosulphate with electrogenerated triiodide and for the titration of ammonia with electrogenerated hypobromite after continuous gas dialytic separation of ammonia from the sample solution.


Journal of Automatic Chemistry, Vol. 15, No. 6 (November–December 1993), pp. 209–216

### A PC-based titrator for flow gradient titrations

**B. Fuhrmann and U. Spohn***

*Martin-Luther-Universität Halle-Wittenberg, Institut für Biotechnologie, Weinbergweg 16a, 06120 Halle/Saale, Germany*

*This paper describes a PC (personal computer) based titrator which was developed for gradient flow titrations. Concentration gradients were generated electrolytically or volumetrically in small tubes. Complete titration curves can be recorded on-line and evaluated automatically. The titrator can be used with all liquid flow detectors with low axial dispersion. The titrator was evaluated for the titration of thiosulphate with electrogenerated triiodide and for the titration of ammonia with electrogenerated hypobromite after continuous gas dialytic separation of ammonia from the sample solution.*

### Introduction

Flow titrations combine the advantages of flow-through analysis (for example high reproducibility, fast mixing, short analysis times and reduced interference from contaminants) with those of titration (high accuracy, precision and reliability). Flow titrations working according to the principle of flow injection analysis have been widely reported [1–4]. There are advantages in this technique (for example high sampling rates and low reagent consumption), but the disadvantages should not be ignored—calibration with a series of standard solutions and non-linearly deformed titration curves which make evaluation difficult.

Programmed coulometric flow titration was developed by Nagy *et al.* in the late 1970s [5–8]; in this linear concentration gradients of the titration reagent are generated electrolytically. Two gradients form a triangle, which is continuously mixed with the sample solution in a dripping vessel. Miniaturization of the flow channel, replacement of the dripping vessel for a coiled tube, and optimization of the titration parameters enables nearly absolute working gradient titrations to be performed in flow channels [9–10].

Valcarcel *et al.* [12 and 13] have used flow rate gradients to generate concentration gradients. They combined a fixed flow rate pump with a programmed flow rate pump. Mixing linear flow rate gradients with constantly flowing solutions resulted in non-linear concentration gradients.

Fuhrmann and Spohn [11] extended the 'triangle' coulometric titration concept to volumetric titrations, and thus extended the technique to more applications. They also proposed the volumetric double gradient titration technique [11] which had an extended determination range. The concentration gradients were generated with two computer-controlled micropumps; the resulting flow rate in the titration tube reactor and the flow detector was held constant enabling nearly absolute working flow titrations to be performed.

The general manifold for triangle programmed gradient flow titrations is shown in figure 1 working with two flow channels, one channel contains the sample of concentration $c_s$ flowing with the rate $\dot{V}_s$ and a triangle mass flow profile of the reagent is propelled through the other channel. The mass flow profile can be described by:

$$\dot{n}_R(t) = \begin{cases} \dot{n}_{R,\max} * t/\tau & 0 < t < \tau \\ \dot{n}_{R,\max}(2 - t/\tau) & \tau < t < 2\tau \end{cases} \quad (1)$$

After mixing the determination reaction:

$$aS + bR \rightarrow \text{products}. \quad (2)$$

takes place in the coil $M$. The flow detector records the corresponding pair of mirror symmetric titration curves. The time difference between the two resulting equivalence points, $t_{eq.,1}$ and $t_{eq.,2}$, depends on the sample concentration.

During coulometric titrations, the reagent mass flow profile is generated electrolytically in a miniaturized flow cell with a low degree of back mixing (see figure 10) by a triangle current programme according to:

$$I(t) = \begin{cases} I_{\max} * t/\tau & 0 < t < \tau \\ I_{\max}(2 - t/\tau) & \tau < t < 2\tau \end{cases} \quad (3)$$

During volumetric titrations, the streams of two computer-controlled pumps are mixed. One pump propels a reagent stock solution, at concentration $c_R$, with the flow rate programme:

$$\dot{V}_R(t) = \begin{cases} \dot{V}_{\max}\, t/\tau & 0 < t < \tau \\ \dot{V}_{\max}(2 - t/\tau) & \tau < t < 2\tau \end{cases}. \quad (4)$$

The other pump propels a diluent with the inverse flow rate programme:

$$\dot{V}_D(t) = \begin{cases} \dot{V}_{\max}(1 - t/\tau) & 0 < t < \tau \\ \dot{V}_{\max}(t/\tau - 1) & \tau < t < 2\tau \end{cases} \quad (5)$$

with a resulting constant total flow rate $\dot{V}_R(t) + \dot{V}_D(t) = \dot{V}_{\max}$.

The object of the work reported here was to develop a fully automated microflow titrator for both coulometric and volumetric titrations.

### Experimental

Figure 2 shows the PC-based flow titrator with its controllable external components. Hardware includes six precision pumps, up to three magnetic valves (Akzo, 368/1/3/24/30, Ratingen, Germany), an electrolysis flow

* *Correspondence to Dr Spohn.*

0142-0453/93 $10.00 © 1993 Taylor & Francis Ltd.

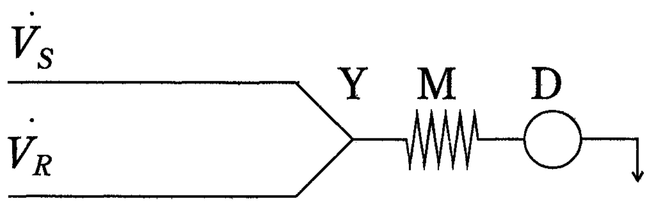

*Figure 1. General manifold for gradient flow titrations; D—flow detector; M—mixing and reaction coil; Y—mixing point; $\dot{V}_S$—sample flow rate; $\dot{V}_R$—reagent flow rate.*

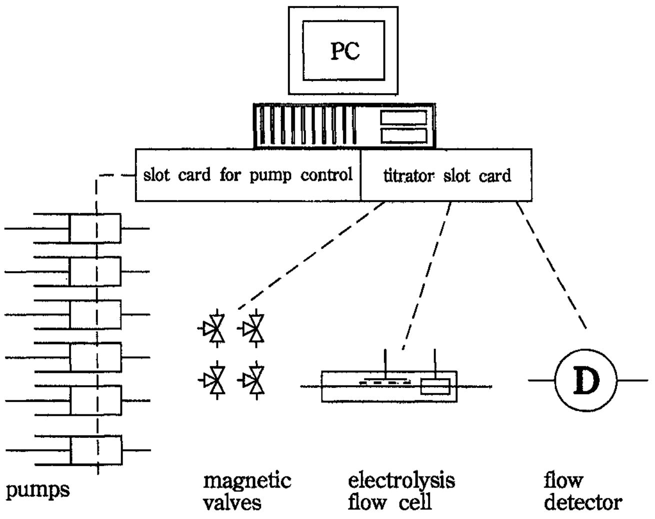

*Figure 2. PC-based flow titrator with all external controllable hardware components.*

cell and a flow detector. A slot card for pump control and the titrator slot card (both developed by the authors), interface and hardware components and the personal computer (IBM-AT). The printed circuit cards were developed following layouts which were designed using a software package called EAGLE [14].

### *Flexible pump control*

The pumps used had to be precise and adjustable; the Dosimat 665 precision piston pumps (Metrohm, Herisau, Switzerland) and TEC-S rotating plunger pumps (Tecuria, Bonaduz, Switzerland) were chosen for the titrator. These pumps can also be used to propel nonaqueous and highly reactive solutions. The rotating plunger pumps have a very small total volume of around 20 µl of the inflow, the pump chamber and the outflow. Both pump are driven by electric impulses. A well-defined volume $V_I$ is propelled per impulse; and the desired flow, $\dot{V}$, is obtained by controlling the frequency, $f_I$, which can be defined as the ratio $f_I = \dot{V}/V_I$.

Figure 3 shows the layout of the pump control card (only one of the six identical channels is included). A quartz clock generator delivers a frequency of 1 MHz and two programmable 16-bit timers (Intel 8253) generate the frequency ($f_i$). The output of the second timer is connected either to the feeding control input of the piston pump or to the stepping motor circuit (SAA 1027, Valvo), which controls the unipolar motors (0·34 A/phase) of the rotating plunger pumps.

A parallel 24 bit input/output circuit (PIO Intel 8255) delivers control signals for the start/stop commands and for changes in the pump direction. The 'ready' signals are fed back to the PIO circuit. The signal output, which initializes the piston filling of the Dosimat 665, is used for changing the flow direction of the rotating plunger pumps. The start/stop output of the PIO is connected to the timer gates and to the start/stop input of the stepping motor control.

To program the timer channels, and the PIO, the data and control busses are connected to those of the PC via the address decoder and the bus buffer. The PIO circuit works in the 0 mode with three independent input/output ports which are defined by the code word 82H. Ports A and C are the output ports and port B is the input port. The timers work in mode 3 as square-wave generators, with binary counting, and enable the gate inputs to be used. The corresponding control word is 36H plus the channel number.

### *The titrator slot card*

Figure 4 shows the titrator slot card, which controls the electrolytic generation of the titration reagents and enables the polarization voltage of the amperometric detection to be held constant and preadjusted by an integrated potentiostat; it also controls magnetic valves.

The indicator circuits for the amperometric detector are galvanically separated from the electrolysis current circuit by insulation amplifiers (ISO122) [15]. The corresponding electrode pairs can be placed in the same flow channel, the digital control signals for the range selection of the electrolysis current, the input selection and the magnetic valve switching are insulated by reed relays and opto-couplers. DC/DC converters HPR 111 [16] were used to insulate the power supply. A 12-bit DAC AD7548 (MAXIM) is directly connected to the address decoder bus buffer for adjustment of the polarization voltage [17]. An output voltage in the range between −10 V and +10 V is generated to use the full range of the insulation amplifier ISO122 to establish the highest possible accuracy. After galvanic insulation the range of polarization voltage is reduced to the range from −1 V to +1 V by an amplification stage of 0·1. The output of that stage is connected to the indicator electrode. The corresponding reference electrode is connected to the input of a current-to-voltage converter, with an amplification factor 2 V/µA. The signal output is connected to input 1 of the analogue multiplexer (HI509, Burr Brown), which can also switch between four analogue sources—this means that external flow detectors can be used. The input voltage range can be scaled over three magnitudes, which are switched over by the instrumentation amplifier INA 120 (Burr Brown), together with the multiplexer HI509. The instrumentation amplifier is followed by an insulated amplifier connected to a 12-bit ADC MAX177 [18] (MAXIM). The ADC is connected to the bus of the Z80 microcomputer.

The electrolysis current is precisely adjusted by a galvanic-insulated and voltage-controlled current source

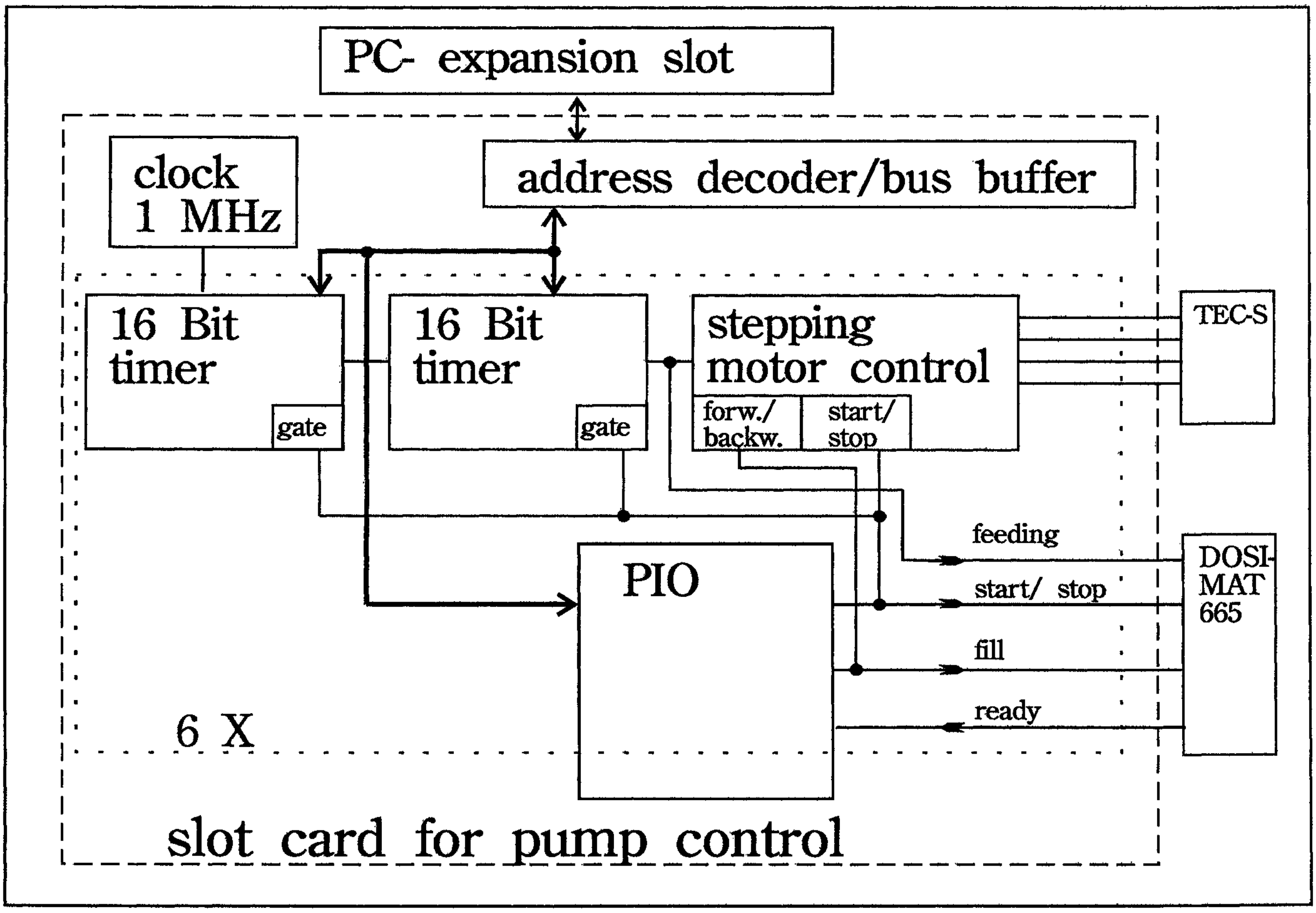

*Figure 3. Slot card for pump control, see text for explanation.*

in three maximum current ranges (2 mA, 100 μA, 5 μA), which is also controlled by the Z80 microcomputer using a 12-bit DAC (AD7548).

The real-time scanning of the detector signal and the generation of precisely defined electrolysis current profiles are controlled by the Z80 microcomputer. It consists of the Z80 CPU, a 32k SRAM (64256), a 32k EPROM (27256), a Z80 CTC and a quartz clock oscillator. A bidirectional latch consists of two 74LS374 circuits, and connects the data bus of the Z80 to the PC. Data and instructions are exchanged in both directions in an interrupt—controlled manner.

*Software*

The control and evaluation software consists of an assembler program for the Z80 processor and a main program for the PC.

*Assembler program*

The assembler program controls the real-time functions of the titrator card and consists of two parts. The first part contains a small operating system and several drivers for the hardware components, which are connected to the Z80 and to the PC for data exchange. This part of the software is stored in the EPROM.

The second part is the firmware, which is started automatically after loading from the PC into the RAM of the Z80 microcomputer. The received parameter block contains the duration and the maximum current of the triangle current profile, the time intervals and the start time of the detector signal recording. The slot card functions can be easily extended and modified by changing the firmware.

The most important tasks of the firmware, which is started from the main program, are:

(1) Transfer of the user defined parameter block from the PC to the Z80.
(2) Generation of current profiles for electrolysis.
(3) Detector signal scanning.

The current profile generation and the detector signal recording work independently of the PC. Both operations are controlled by timer interrupts from the Z80CTC. The recorded detector signal can be sent in real time to the PC or stored in the Z80's RAM. If stored, then the detector signal values can be sent later to the PC for further signal course evaluaton.

*Main program*

The main program, CEFT, is structured according to the scheme shown in figure 5. The user can freely configure the titrator from the main menu. Table 1 shows the

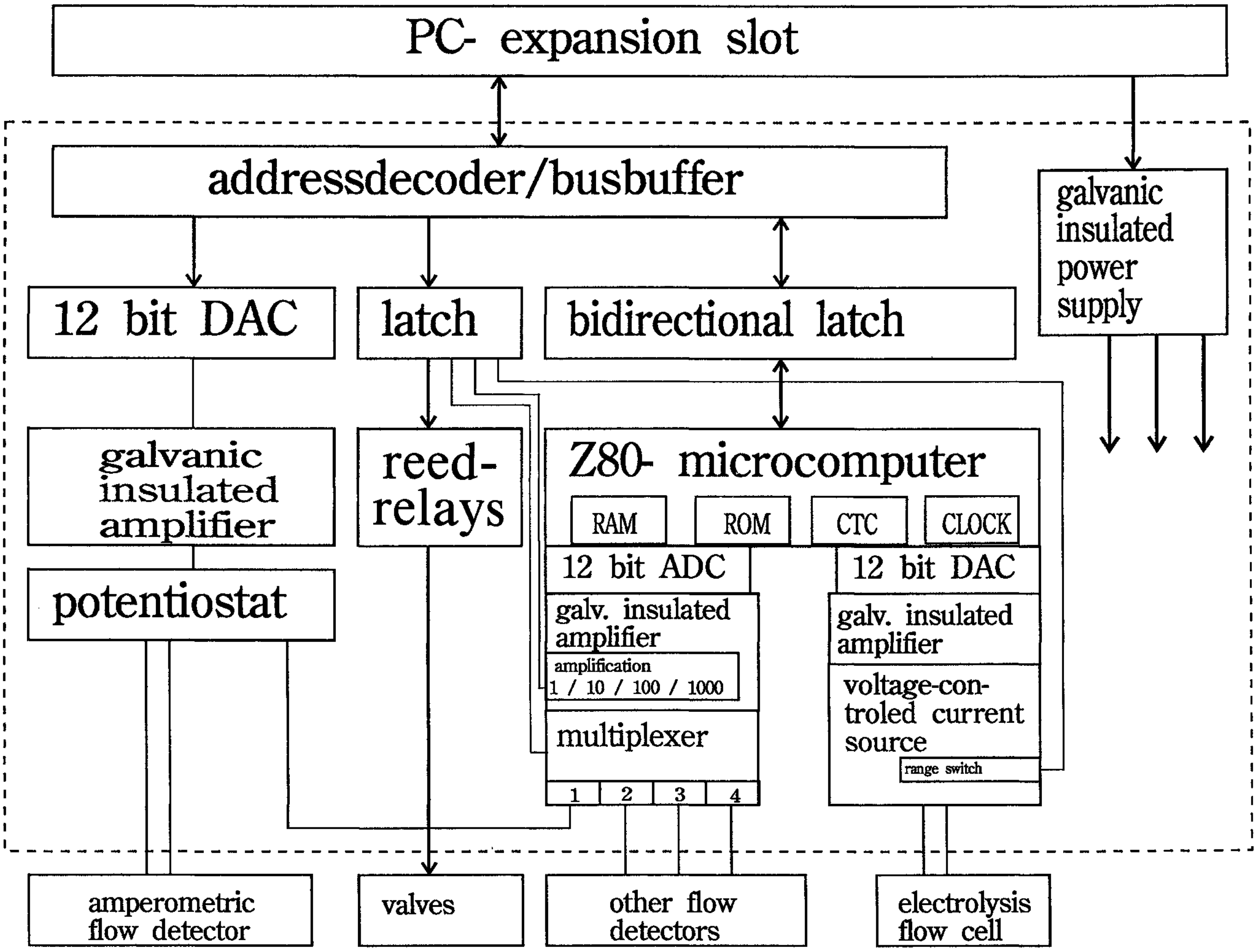

*Figure 4. Titrator slot card, see text for explanation.*

*Table 1. Flow titration modes and parameters.*

| | |
|---|---|
| Titration mode | |
| volumetric titration | —single gradient<br>—double gradient<br>—back titration |
| coulometric titration | —direct titration<br>—back titration |
| Titration parameters | |
| Volumetric titration | —stoichiometric factors of the titration reaction<br>—maximum reagent concentration |
| Coulometric titration | —number of electrons per electrolytically generated ion or molecule<br>—maximum electrolysis current<br>—stoichiometric factors of the titration reaction |
| Control parameters | —step volume $V_i$ for each pump channel<br>—number and concentrations of standard solutions<br>—polarization voltage for the amperometric flow detector<br>—pump rates<br>—analogue input channel for the detector |

parameters and control modes which can be selected by the user.

The system offers three automatic calibration modes:

(1) With precalculated working lines.
(2) With standard solutions.
(3) With memorized calibration lines.

As demonstrated earlier [11], the precalculated lines can be used for calibration after adjustment of optimum parameter values. The analytical results, $c_S$, are calculated from the measured time differences between the two on-line evaluated equivalence points, $t_{eq,1}$ and $t_{eq,2}$ (see table 2). The index max signs the parameter values at $t = \tau$. The maximum value of the mass flow $\dot{n}_{R,\max}$ is related to the maximum electrolysis current and to the maximum flow rate $\dot{V}_{R,\max}$.

Figure 6 is a flow chart of the fully automatic procedure with $N$ user-defined standard solutions and $m$ titrations per standard concentration value. The standard solutions are automatically produced *in situ* with two precision pumps. For every preadjusted concentration, $c_i$, the flow rates $\dot{V}_{St,i}$ and $\dot{V}_{D,i}$ of the standard stock solution, $St$, with the concentration $c_{St}$ and of the diluent $D$, respectively are calculated with the flow rate $\dot{V}_S$, which is preadjusted

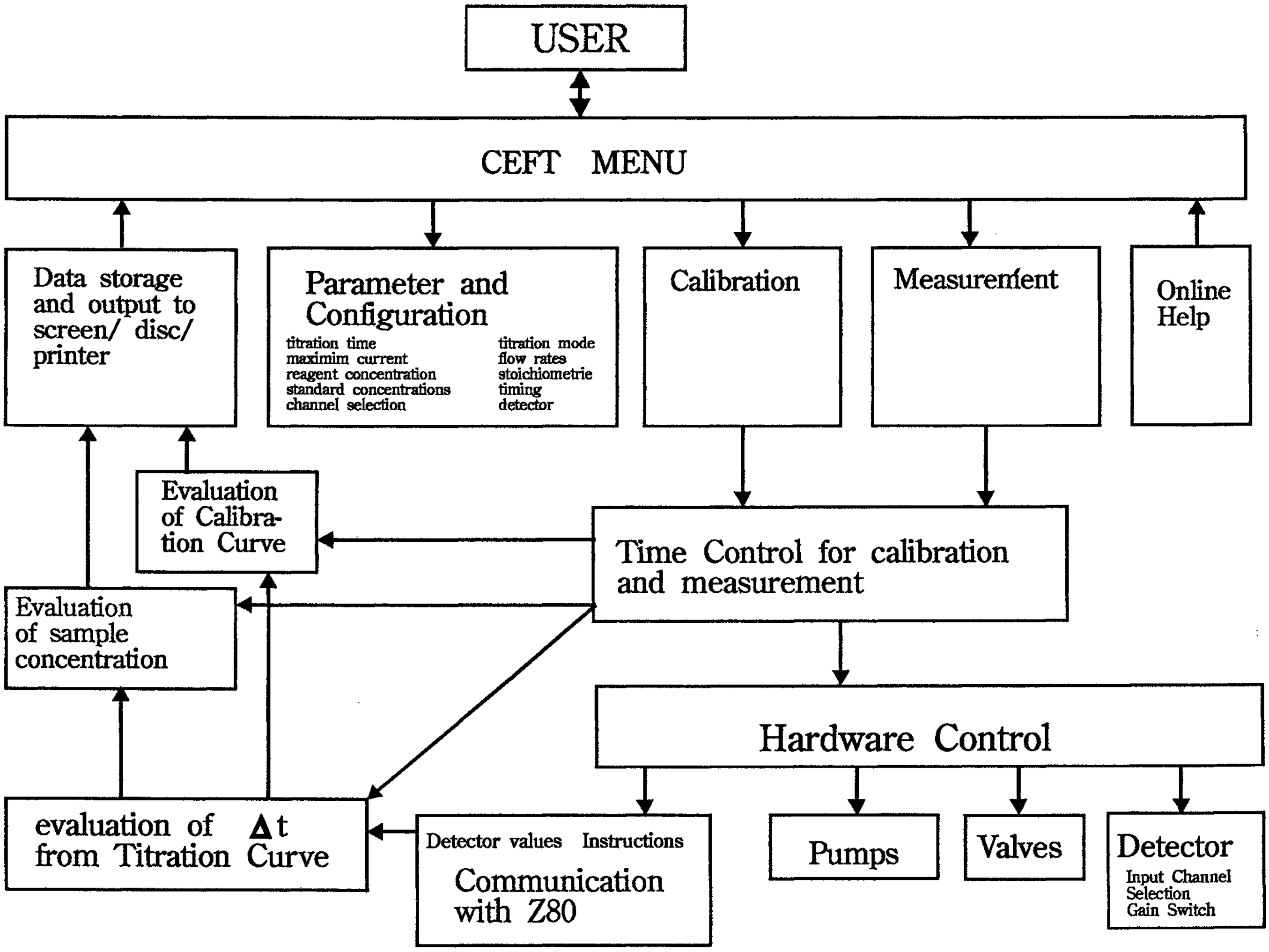

*Figure 5. Internal structure of the main program for the titrator, see text for explanation.*

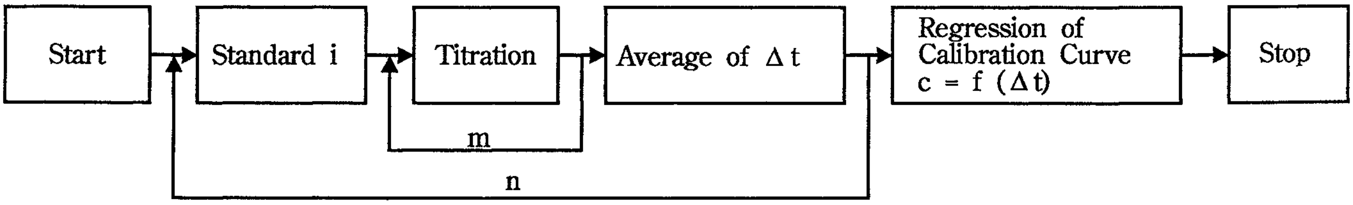

*Figure 6. Flow scheme of the automatic self-calibration.*

also for the sample solution, according to

$$\dot{V}_{St,i} = c_i/c_{St,i} * \dot{V}_S \quad (9)$$

and

$$\dot{V}_{D,i} = \dot{V}_S - \dot{V}_{St,i}. \quad (10)$$

Thereafter $m$ titrations are automatically implemented for every standard concentration $c_i$. Then the calibration line is calculated by regression analysis. The calibration results and the relevant titration parameters are saved in a calibration file.

Previously saved calibration curves can be loaded in many cases the important parameters, for example the pump rates and the electrolysis current, can be held constant over long time periods. Coulometric titrations and volumetric titrations with stable titration reagents have calibration lines with a high long-term stability.

After calibration, the analysis of sample solutions is carried out. The user can preselect the number and the time period of the titrations. The results can be printed or stored in an ASCII-file after each titration. Figure 7 shows the calculation of $\Delta t$ from the titration curves. First, a regression line for the base-line is calculated; then the signal height of the curve relative to the base-line is determined. The linear segments of both titration curves are automatically selected on the basis of user-defined upper and lower signal levels, with respect to the maximum signal heights. The data points in the resulting signal window are used to calculate regression lines for the ascending and descending parts of the titration curves.

*Table 2. Theoretical working lines.*

| | | |
|---|---|---|
| Direct titration | $c_S = \left(1 - \frac{\Delta t}{2\tau}\right)\frac{a}{b}\frac{\dot{n}_{R,\max}}{\dot{V}_S}$ | (6) |
| Back titration | $c_S = \frac{1}{\dot{V}_S}\frac{c}{d}\left[c_U \dot{V}_U - \left(1 - \frac{\Delta t}{2\tau}\right)\dot{n}_{R,\max}\right]$ | (7) |
| Volumetric double gradient | $c_S = \left(\frac{2\tau}{\Delta t} - 1\right)\frac{a}{b} c_R$ | (8) |
| Reagent profile generation | | |
| Coulometric | $\dot{n}_{R,\max} = \frac{I_{\max}}{Fz}$ | |
| Volumetric | $\dot{n}_{R,\max} = c_R \dot{V}_{R,\max}$ | |

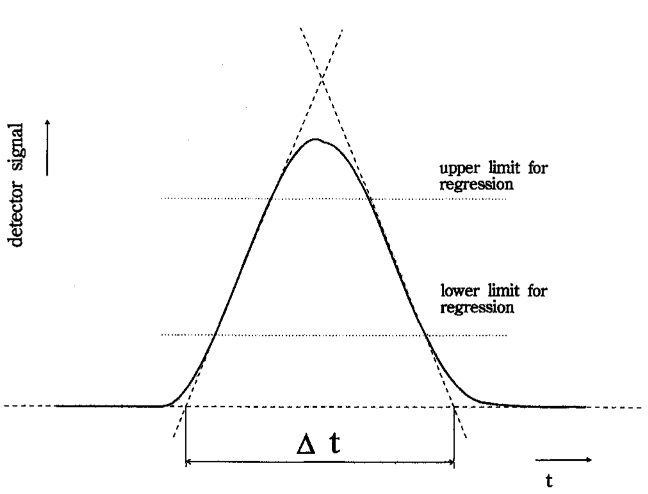

*Figure 7. Measurement and calculation of $\Delta t$, see text for explanation.*

The time difference, $\Delta t$, is finally calculated as the time difference between the points of intersection with the base-line.

### Results and discussion

Table 3 shows the flow titrator's working parameters. Sampling rate and sample consumption depend on the tiration time $2\tau$.

The electrolysis current source was tested with working resistors in the range between 0.1 and 47k Ohm. The relative deviation of the measured from the preadjusted current was not greater than 0·2% in the range between 0·05–2 mA, and smaller than 0·5% in the range 0·05–5 µA. The time interval between two changes of electrolysis current is adapted automatically to the titration time $2\tau$ and the maximum electrolysis current $I_{\max}$ in the range from 1·25 ms to $2^{16} * 1{\cdot}25$ ms, so that the maximum number of current steps approximates a linear time function of the electrolysis current.

*Table 3.*

| Parameter | Ranges |
|---|---|
| Electrolysis current | 0·5 µA–2 mA in the three $I_{\max}$ ranges between 0 and 2 mA, 100 µA and 5 µA, respectively, with a resolution of 12 bit |
| Titration time | coulometric titrations: $2\tau > 10$ s<br>volumetric titrations: $2\tau > 50$ s |
| Flow rates | 0·05–0·8 ml/min for the TEC-S pumps<br>0·05–10 ml/min for the Dosimates 665 |
| Sample consumption | 0·1–1·5 ml/min |
| Sampling rate | >30/h |

For the generation of volumetric concentration gradients, the time between two changes of the flow rate is adapted to $2\tau$. The shortest time step is 0·1 s. The generation of nonlinear concentration gradients can also be programmed.

The flow rates can be adjusted with a relative standard deviation of 0·15% ($\mathcal{N} = 5$, $\alpha = 0{\cdot}05$) for the piston pumps and 0·5% for the rotating plunger pumps. Since two 16-bit counters are used to generate the frequency $f_I$, the digital resolution does not limit the precision of the pumps.

The detector signal is measured 10 times/s. The precision of the equivalence point determination depends on the slopes of the ascending and descending parts of the titration curves. Standard deviations between 0·2 and 0·5 s ($\mathcal{N} = 5$, $\alpha = 0{\cdot}05$) can be achieved.

The flow titrator can be used for a variety of volumetric and coulometric titrations: table 4 summarizes tested titration procedures.

Recovery was better than 99% in all cases ($\mathcal{N} = 4$, $\alpha = 0{\cdot}05$). The corresponding relative standard deviations were between 0·2 and 2% ($\mathcal{N} = 4$, $\alpha = 0{\cdot}05$).

Figure 8 shows a series of biamperometric flow titration curves for different thiosulphate concentrations, which

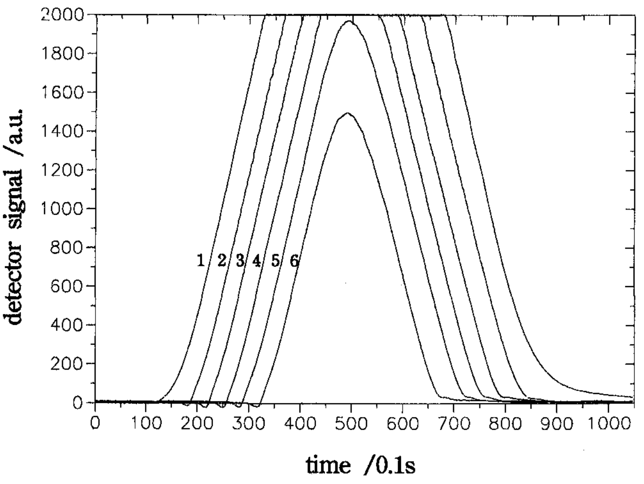

*Figure 8. Titration of thiosulphate with electrogenerated triiodide; biamperometric titration curves, 1–6; $c_S$ = 0, 0·04, 0·08, 0·12, 0·16, 0·2 mmoll$^{-1}$; $2\tau = 69{\cdot}8$ s; $V_s = 0{\cdot}5$ ml min$^{-1}$; $I_{\max} = 0{\cdot}3$ mA.*

*Table 4.*

| | Sample | reagent | Range/limit of detection | Detection |
|---|---|---|---|---|
| Volumetric | HCl | NaOH | 0·1–100 mM/0·01 mM | photometric with bromthymol blue/11/ |
| | pyruvate | $NADH^{(1)}$ | 0·01–1 mM/0·002 mM | fluorimetric |
| Coulometric titrations | $HSO^{3-}$ | $I^{3-(2)}$ | $5*10^{-7}$–$10^{-3}$ M/$10^{-7}$ M | biamperometric |
| | $S^2$–$S_2O_3{}^{2-}$ | $I^{3-}$ | $3*10^{-7}$–$10^{-3}$ M/$10^{-7}$ M | biamperometric |
| | $OBr^-$ | $NH_3{}^{(3)}$ | $10^{-6}$–$5*10^{-3}$ M/$10^{-6}$ M | amperometric |
| | $OH^-$ | $HCO_3{}^{2-}$ | $10^{-5}$–$5*10^{-3}$ M<br>$5*10^{-6}$ M | photometric |

LDH—Lactate dehydrogenase, (1) catalyzed by lactate dehydrogenase, (2) after gasdialytic separation in the set-up shown in figure 9, (3) see text for explanation.

were titrated with electrogenerated triiodide in a continuously flowing electrolyte (0·1 M KI, 0·1 M KCl). The biamperometric detector consists of two identical platinum tube electrodes with an inner diameter of 0·5 mm and a length of 5 mm. A constant polarizing voltage of 100 mV was applied between the electrodes; the electrodes were separated by a Teflon ring which was of 1 mm thick. Comparing the precalculated (11) and the measured (12) working lines:

$$c_S = 0{\cdot}373\ \text{mmoll}^{-1} - 5{\cdot}36\ \mu\text{moll}^{-1}\,\text{s}^{-1} * \Delta t \qquad (11)$$

$$c_S = 0{\cdot}384\ \text{mmoll}^{-1} - 5{\cdot}56\ \mu\text{moll}^{-1}\,\text{s}^{-1} * \Delta t \qquad (12)$$

demonstrates that nearly absolute determinations are possible for thiosulphate concentrations between 0·01 mM and 0.25 mM. Sulphite and sulphide can also be titrated with electrogenerated triiodide in the same titration set-up and under the same conditions.

The advantages of the described flow titrator were also demonstrated for the coulometric titration of ammonia, after gasdialytic separation from sample solutions with electrogenerated hypobromite according to:

$$2NH_3 + 3OBr^- \rightarrow N_2 + 3Br^- + 3H_2O. \qquad (13)$$

Figure 9 shows the set up, which consists of a gas dialysis cell, GD; a flow-through electrolysis cell, EC; a biamperometric flow detector, BFD; and a mixing and reaction coil, M. These are connected by Teflon tubes with an inner diameter of 0·5 mm; all solutions are propelled by miniaturized rotating plunger pumps: P1–P4.

Figure 10 illustrates the flow electrolysis cell. The generator electrode chamber and the auxiliary electrode chamber are separated by a cation exchange membrane—

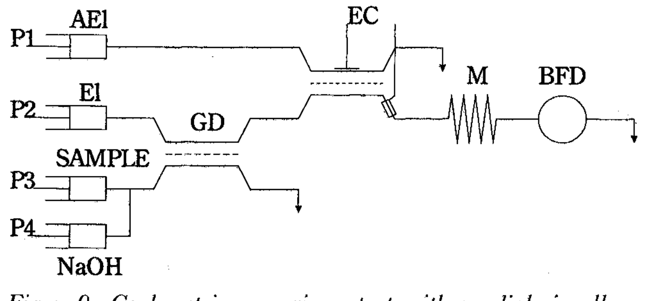

*Figure 9. Coulometric measuring set-up with gas dialysis cell; see text for explanation.*

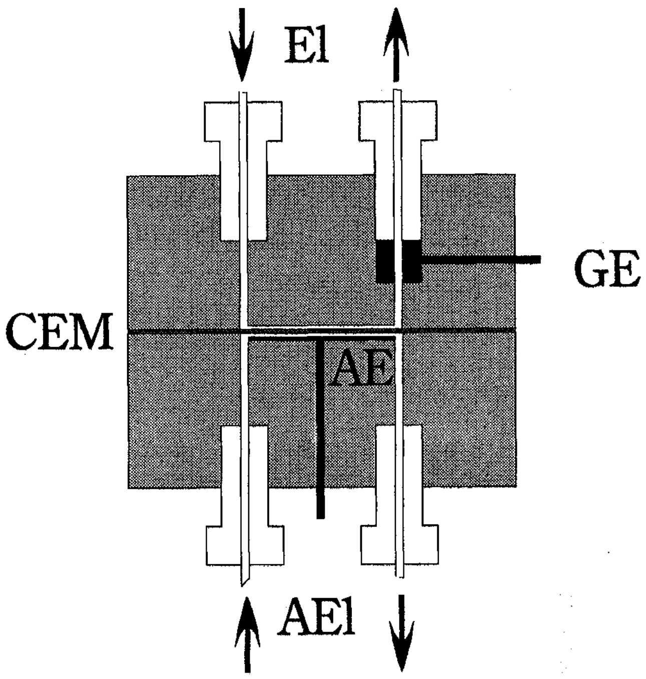

*Figure 10. Flow electrolysis cell for the generation of titration reagents; see text for explanation.*

CEM (NAFION 127, DuPont Nemours, USA). The auxiliary electrode, AE, is a Platinum (Pt) foil with an accessible area of 15 $mm^2$. A Pt-tube, with an inner diameter of 0·5 mm and a length of 5 mm, is the generator electrode GE. Both the auxiliary electrolyte (AEl) and the working electrolyte (El) contain 0·2 KBr and 0·1 M $NaHCO_3$ (pH 8·3).

Figure 11 shows the gas dialysis cell: this separation cell consists of two mirror symmetric KelF-plates with groove labyrinths (groove width 1·5 mm, depth 0·2 mm). A microporous teflon membrane, which has a mean pore size of 0·2 μm and is 20 μm thick (from Sartorius, Göttingen, Germany), is mounted between the two plates. The membrane exchange area is 505 $mm^2$.

To determine the sum of ammonia and ammonium, the sample solution is mixed with a 0·2 M NaOH solution to convert ammonium completely into the volatile ammonia. Between 0·1 mM and 2 mM ammonia, the calibration graph (14) is almost identical to the precalculated

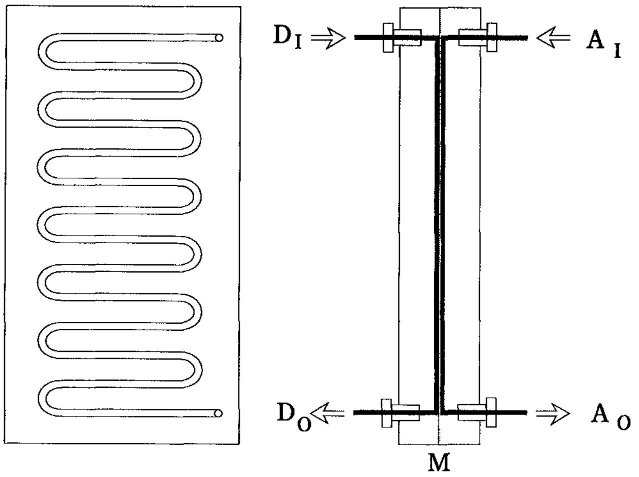

*Figure 11. Gas dialysis cell, $D_I$, $D_O$: donor in and out; $A_I$, $A_O$: acceptor in and out.*

line (15):

$$cS = (3{\cdot}42 \pm 0{\cdot}02)\ \text{mmoll}^{-1} - (0{\cdot}04 \pm 0{\cdot}04)\ \text{mmol}^{-1}\ \text{s}^{-1} * \Delta t \quad (14)$$

(where $n = 4$, $\alpha = 0{\cdot}05$, $r^2 = 0{\cdot}9998$)

$$cS = 3{\cdot}45\ \text{mmoll}^{-1} - 0{\cdot}04\ \text{mmol}^{-1}\ \text{s}^{-1} * \Delta t. \quad (15)$$

Separation efficiency can be calculated from the slope factors to be (99·1 ± 0·72)%. The ammonia determination is not disturbed by non-volatile interferences, which are oxidized by hypobromite, for example thiosulphate, sulphite, cyanide, urea and glutamine with concentrations not greater than 50 mM. Greater concentrations can influence the activity coefficients of ammonia and ammonium. The working parameters are $\dot{V}_K = 0{\cdot}05$ ml $\text{min}^{-1}$, $\tau = 78{\cdot}1$ s and $I_{max} = 2{\cdot}5$ mA.

The combination of the coulometric flow titration of ammonia with a complete analyte separation by gas dialysis and with enzyme reactors, which convert all of the analyte, enables nearly absolute determinations of glutamine and urea to be performed [10]. The titrator was used for simultaneous on-line determinations of ammonia and glutamine in animal cell culture media [19].

### Conclusions

The PC-based microflow titrator described in this paper enables volumetric and coulometric titrations to be performed. Fast and precise flow titrations are possible, covering a wide range of applications. Titration is in small tubes (with volumes smaller than 100 µl) so much less of the reagent and sample solutions is used in comparison with conventional titrations. Short titration times (between 10 and 120 s) and short rinsing times are other advantages, especially for process analysis applications.

The selectivity of flow titrations can be increased with continuously working gas dialysis separation to determine volatile analytes.

In addition, other flow detectors can be used to extend the application field fo the PC-based flow titrator.

### References

1. Ruzicka, J., Hansen, E. H. and Mosbaek, H., *Analytica Chimica Acta*, **93** (1977), 235.
2. Ramsing, A. U., Ruzicka, J. and Hansen, E. H., *Analytica Chimica Acta*, **129** (1981), 1.
3. Koupparis, M. A., Anagnostopoulou, P. and Malmstadt, H. V., *Talanta*, **32** (1985), 411.
4. Tyson, J. F., *Analyst*, **112** (1987), 523.
5. Nagy, G., Feher, Zs., Toth, K. and Pungor, E., *Analytica Chimica Acta*, **91** (1977), 87.
6. Nagy, G., Feher, Zs., Toth, K. and Pungor, E., *Analytica Chimica Acta*, **91** (1977), 97.
7. Nagy, G., Feher, Zs., Toth, K. and Pungor, E., *Analytica Chimica Acta*, **100** (1978), 181.
8. Nagy, G., Toth, K. and Pungor, E., *Analytical Chemistry*, **47** (1975), 1460.
9. Spohn, U., Nagy, G. and Pungor, E., *Analytical Science*, **2** (1986), 423.
10. Fuhrmann, B. and Spohn, U., *Biosensors and Bioelectronics*, **7** (1992), 653.
11. Fuhrmann, B. and Spohn, U., *Analytical Chimica Acta*, **282** (1993), 397.
12. Marcos, J., Rios, A. and Valcarcel, M., *Analytical Chimica Acta*, **261** (1992), 489.
13. Marcos, J., Rios, A. and Valcarcel, M., *Analytica. Chimica Acta*, **261** (1992), 495.
14. *EAGLE Handbook*, Cadsoft Computer GmbH, Rosenweg 42, D(W) 8261, Pleiskirchen, Germany.
15. Technical Documentation, ISO 122P Precision Lowdst Cost Isolation Amplifier, Burr-Brown, PO Box 11400 Tucson, Arizona 85734, USA (1990).
16. Technical Documentation, HPR1XX Series DC/DC converters, Burr-Brown, USA (1990).
17. Technical Documentation, CMOS 8-Bit µP-Compatible 12-Bit DAC 120, MAXIM Integrated Products, San Gabriel Drive, Sunnyvale, California 94086, USA (1989).
18. New Releases Data Book, 120 MAXIM Integrated Products, San Gabriel Drive, Sunnyvale, California 94086, USA (1992).
19. Fuhrmann, B., Spohn, U., Mohr, K. H. and Wandrey, Chr., 2nd Bioelectroanalytical Symposium (Akademiai Kiado, Budapest, 1992), 385.
20. Fuhrmann, B., Ph.D. thesis, University of Halle (1994).

### Appendix

*Symbols used in the text*

| | |
|---|---|
| $c_S$, $c_R$, $c_U$: | sample, reagent and excess reagent concentrations in $\text{moll}^{-1}$. |
| $\dot{V}_S$, $\dot{V}_R$, $\dot{V}_U$: | Sample, reagent and excess reagent flow rates in $\text{l s}^{-1}$. |
| $a$, $b$, $c$, $d$: | Stoichiometric factors of determination and back reactions. |
| $2\tau$: | Titration time in s. |
| $I$: | Electrolysis current in A. |
| $z$: | Number of electrons for the generation of one ion or one molecule of the titration reagent. |
| $F$: | Faraday's constant. |

